# Research progress on the fibrinolytic enzymes produced from traditional fermented foods

**DOI:** 10.1002/fsn3.3601

**Published:** 2023-08-07

**Authors:** Panpan Wang, Cuiying Peng, Xiaomei Xie, Xiongwei Deng, Meizhi Weng

**Affiliations:** ^1^ Top Discipline of Jiangxi Province, Discipline of Chinese and Western Integrative Medicine Jiangxi University of Chinese Medicine Nanchang China; ^2^ Nanchang Hongdu Hospital of TCM Affiliated to Jiangxi University of Chinese Medicine Nanchang China

**Keywords:** cardiovascular diseases, fibrinolytic enzymes, functional properties, thrombolysis, traditional fermented foods

## Abstract

Cardiovascular diseases (CVDs) are a global health problem and leading cause of death worldwide. Thrombus formation, one of the CVDs, is essentially the formation of fibrin clots. The existing thrombolytic agents have the disadvantages of high price, short half‐life, and high bleeding risk; hence, there is an urgent need to find the alternative thrombolytic agents. In recent years, traditional fermented foods have been widely investigated for their outstanding effects in the prevention and treatment of thrombus formation. In this review, we have focused on fibrinolytic enzymes produced by microorganisms during the fermentation of traditional fermented foods and their potential use for treating CVDs. First, we discussed about the sources of fibrinolytic enzymes and microbial strains that produce those enzymes followed by the optimization of fermentation process, purification, and physicochemical properties of fibrinolytic enzymes. Finally, we have summarized the thrombolytic effects of fibrinolytic enzymes in humans and mice. Fibrinolytic enzymes produced by microorganisms during the fermentation of traditional fermented foods not only lyse thrombi but also acts as anti‐atherosclerotic, anti‐hyperlipidemia, and neuroprotection agents. Therefore, fibrinolytic enzymes from traditional fermented foods have great potential for the prevention and treatment of CVDs.

## Graphical abstract

This article focuses on fibrinolytic enzymes produced by microorganisms during the fermentation of traditional fermented foods. First, we provide an overview of the sources of fibrinolytic enzymes, including strains that produce such enzymes. Then, we discuss the optimization of fermentation process, purification, and physicochemical properties of fibrinolytic enzymes separately. Finally, we summarize the thrombolytic effects of fibrinolytic enzymes in humans and mice, respectively.

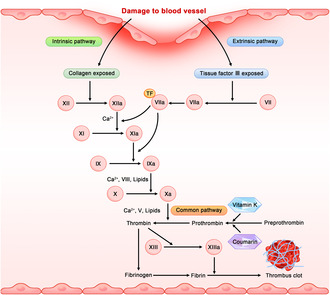

## 1 INTRODUCTION

**FIGURE 1:**
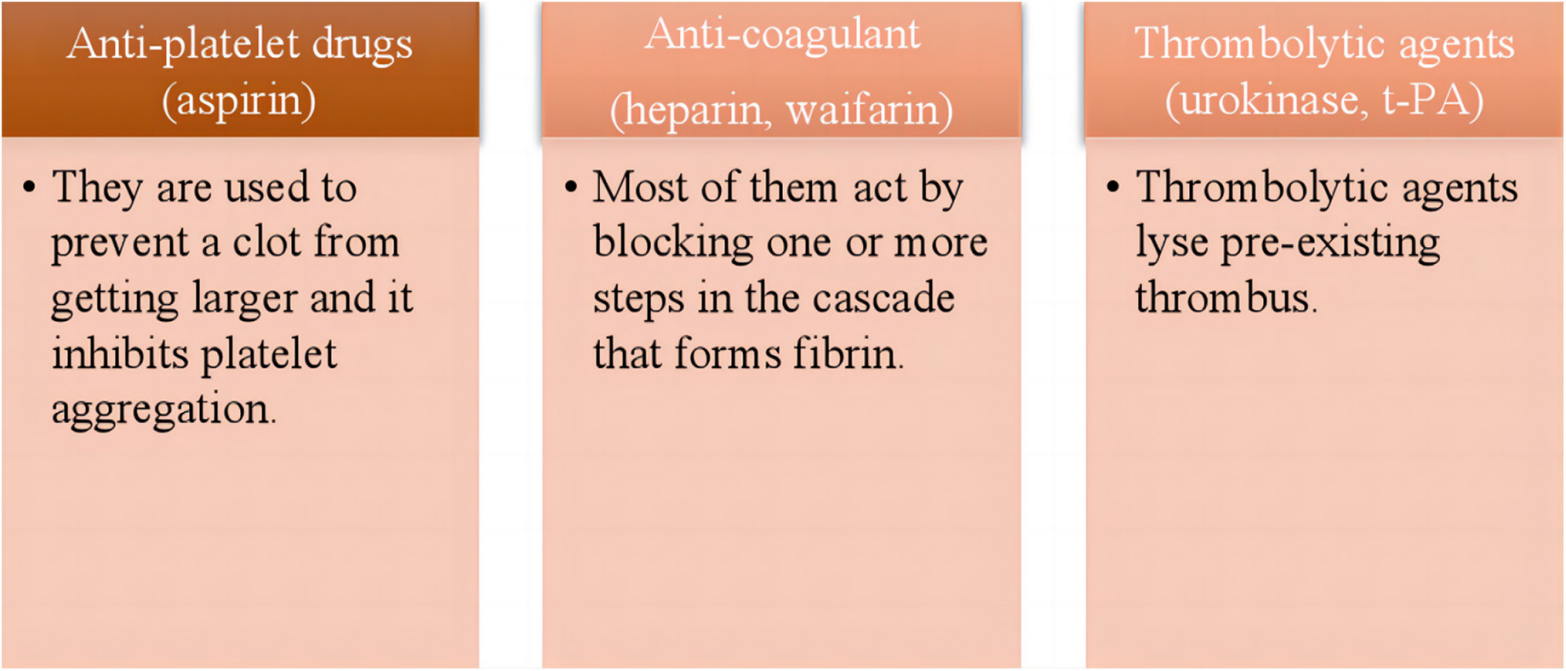
Commonly used anti‐platelet aggregation, anti‐coagulant, thrombolytic agents, and mechanisms of action.

Cardiovascular diseases (CVDs) are a leading cause of deaths worldwide. According to “Global Burden of Cardiovascular Diseases and Risk Factors: 1990–2019,” the number of deaths has increased from 12.1 million to 18.6 million (1990–2019), and an estimated number of 23.3 million people will be affected by 2030 (Roth et al., 2020; World Health Organization, 2016). Angina pectoris and myocardial infarction occur in severe intracerebral hemorrhage (ICH), and the formation of fibrin clots in the blood vessels is one of the major contributing factors to it (Sharma et al., 2021). Fibrin clots, the main component of a thrombus, are formed from fibrinogen that is catalyzed by thrombin. Under normal physiological conditions, there exists a balance between the formation and degradation of fibrinogen. When the balance of the clotting system and the fibrinolytic system is broken, fibrinogen accumulates, leading to the formation of fibrin clots (Wong & Mine, 2004). Currently, the common types of drugs used for clinical intervention include platelet aggregation inhibitors (aspirin), anti‐coagulants (heparin and warfarin), and thrombolytic agents (Barzkar et al., 2022; De Carlo et al., 2023) (Figure 1). However, clinical data revealed that treatments with these drugs were associated with substantially increased risk of complications, including cerebral hemorrhage (Christopoulou et al., 2017; Dimitriadis et al., 2022; Geller et al., 2023; Reish et al., 2023; Tanaka et al., 2023). Therefore, safer and more effective methods to prevent the thrombus formation are required.

As the industrial production mode of fibrinolytic enzymes is evolving, production of fibrinolytic enzymes from traditional fermented foods has become a research focus of many enterprises. Over the years, many foods derived from fibrinolytic enzymes have been found in a variety of traditional Asian foods (Peng et al., 2003; Weng et al., 2017; Yang et al., 2020). During the fermentation process, microorganisms produce various enzymes that have been isolated, purified, and characterized by salting out of proteins using ammonium sulfate precipitation, ion‐exchange chromatography, gel‐filtration (GF) chromatography, and assay of fibrinolytic activity (D'Souza et al., 2020; Hu et al., 2019; Kim et al., 2020).

Therefore, based on these findings, the current article reviews about microbial fibrinolytic enzymes derived from traditional fermented foods and discusses sources, optimization of fermentation process, isolation and purification, physicochemical properties, and thrombolytic potential in vivo. We have also included the insights on serious challenges to produce fibrinolytic enzymes from traditional fermented foods and their application prospect.

## 2 FIBRINOGEN AND MECHANISM OF THROMBUS FORMATION

Fibrinogen (Fg) is the main component implicated in thrombus formation, and its lysis is critically important for revascularization. Therefore, it is necessary to address the underlying mechanisms of thrombus formation and fibrinogenolysis before discussing fibrinolytic enzymes derived from fermented foods.

**FIGURE 2:**
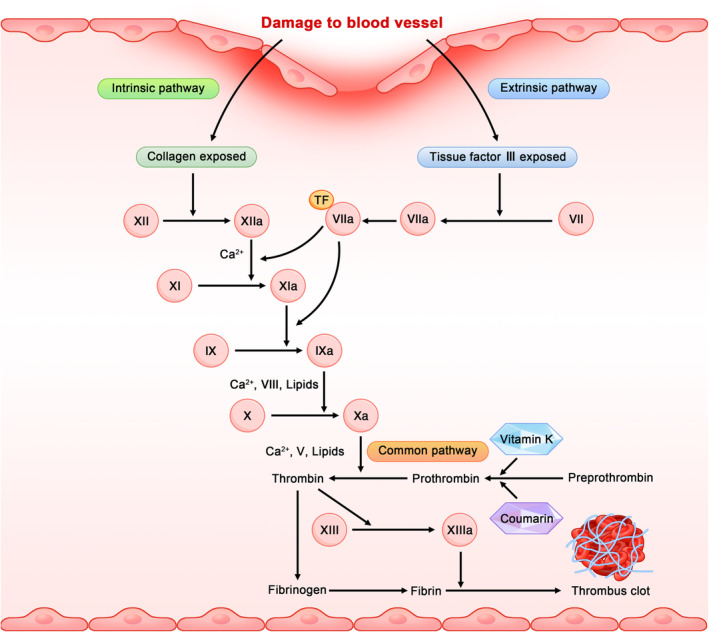
Coagulation cascade and mechanism of thrombus formation. Three pathways, namely extrinsic, intrinsic, and common, are jointly implicated in the occurrence of coagulation cascade, during which the activation of coagulation factors is accomplished, eventually leading to formation of blood clots. The final step is to stabilize the blood clots to prevent excessive loss of blood from the body. The intrinsic pathway is triggered by exposed collagen to activate each coagulation factor in a cascade manner. On the contrary, upon a tissue injury, coagulation factor III is released and the extrinsic pathway is simultaneously triggered.

Thrombus formation is closely involved in blood clotting process in the heart or blood vessels of a living body. Upon damage to vascular endothelial cells, collagen exposure, release of von Willebrand factor (vwf), and tissue factors jointly initiate the coagulation system. Coagulation is a complicated process that includes three enzyme‐catalyzed reaction cascades: intrinsic, extrinsic, and common (Figure 2) (Chapin & Hajjar, 2015; Klein et al., 2012). Activation of the intrinsic pathway of coagulation is triggered by collagen exposed to the endothelium, converting inactivated coagulation factor XII to activated XIIa and consequently activating factor XI to XIa. In the presence of Ca^2+^, activated factor IX activates coagulation factor X with factor VIII, leading to the generation of more thrombin downstream (Moula Ali & Bavisetty, 2020). The extrinsic pathway is initiated by the release of tissue factor III into the bloodstream and activates fewer factors than the intrinsic pathway. The common pathway mainly consists of two processes: the formation of thrombin and the stabilization of fibrin clots. Factor Xa formed via the extrinsic or intrinsic pathway activates prothrombin to thrombin; activated thrombin binds to the N‐terminal of the Aα and Bβ chains of Fg E‐region, removing the N‐terminal fibrino‐peptides to convert to fibrin monomers. The newly exposed α‐ and β‐binding sites are inserted into Hole A and Hole B, respectively, at the γ and β chain C‐terminal of another fibrin monomer in the D region, resulting in half‐staggered binding of fibrin monomers into fibrils (Undas & Ariëns, 2011; Ząbczyk & Undas, 2017). Subsequently, in the presence of thrombin and factor XIII, the fibrils spontaneously cluster into reticular soluble proteins and adhere to activated platelets in the injured blood vessels to form a firm thrombus.

## 3 PLASMIN AND FIBRINOLYTIC SYSTEM

**FIGURE 3:**
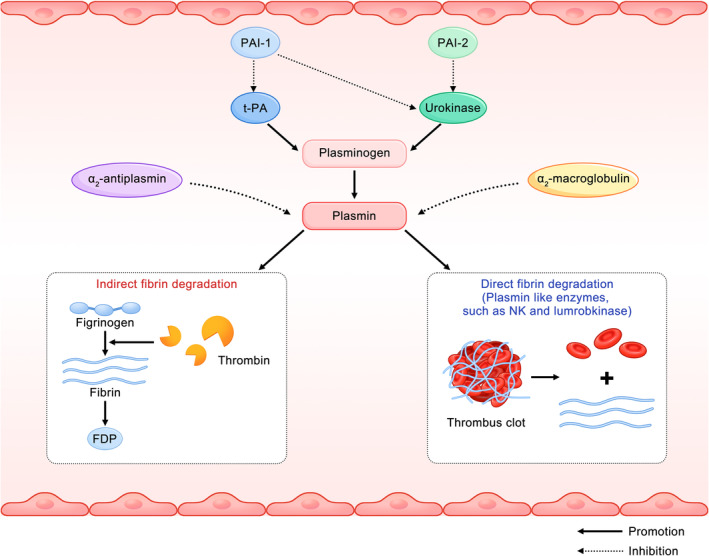
Fibrinolytic system and mechanism of thrombus degradation. The fibrinolytic system mainly includes two processes: activation of plasminogen and degradation of fibrin. In the presence of physiological activators t‐PA and urokinase, the inactive plasminogen is transformed into an active fibrinolytic enzyme to degrade fibrinogen. The process in which thrombin acts on fibrinogen to produce the breakdown product FDP is called indirect thrombolysis. As shown in the figure, NK and lumbrokinase dissolve the thrombus by acting on the blood clots directly. In addition, plasminogen activation is subject to inhibition by plasminogen activator inhibitor‐1 (PAI‐1) and plasminogen activator inhibitor‐2 (PAI‐2), and plasmin is inhibited by ɑ2‐antiplasmin and ɑ2‐macroglobulin.

Plasmin is a proteolytic enzyme that lyses fibrin. In the body, the coagulation and fibrinolytic systems are mutually dependent and closely related. Once a coagulation reaction occurs in the body, the fibrinolytic system is also activated spontaneously and reduces the fibrinogen levels through negative feedback, thus avoiding excessive fibrin agglomeration (Collet et al., 2006). Fibrinolysis can occur through two different mechanisms: (1) indirect fibrinolysis controlled by activation of plasminogen to plasmin and (2) direct fibrinolysis caused by plasmin‐like enzymes. On the one hand, the direct mechanism contains plasmin‐like enzymes, such as nattokinase (NK) and lumbrokinase, which can directly degrade the reticular fibrin. On the other hand, the indirect mechanism is the activation of plasminogen to plasmin, which in turn leads to the cleavage of fibrin, ultimately producing soluble fibrin degradation products (FDPs) (Kumar & Sabu, 2019) (Figure 3). In addition, tissue‐type plasminogen activator (t‐PA) and urokinase‐type plasminogen activator (uPA) are both physiological activators of plasminogen, while α2‐antiplasmin and α2‐macroglobulin are plasmin inhibitors that terminate fibrinolysis. They all work synergistically to maintain the balance of the fibrinolytic system and avoid excessive lysis of fibrin or fibrinogen that leads to bleeding tendency in the body (Chang et al., 2020; Kanno & Shu, 2022).

## 4 SOURCES OF FIBRINOLYTIC ENZYMES

In the last decades, many fibrinolytic enzymes from natural sources have been identified, such as those extracted from the snake venom, earthworm, vampire bats, and microorganisms (Kazemali et al., 2014; Mihara et al., 1991; Sharma et al., 2021; Silva et al., 2003). Microbial fibrinolytic enzymes, particularly those derived from traditional fermented foods are promising sources of thrombolytic agents due to their low production cost, rich species, and achievable genetic engineering. During food fermentation, fibrinolytic enzymes produced from microbial strains are the main functional component to dissolve thrombus. As shown in Table 1, it can be found that there are many types of fermented foods, mainly fermented soy products, such as Pigeon pea, Natto, Douchi, and Korean Cheonggukjang. Due to their high nutritional value and established abilities to dissolve fibrin, it can be used to prevent and treat cardiovascular diseases. These fibrinolytic enzymes are a popular focus of research on cardiovascular diseases in recent years (Lee et al., 2015). It can also be concluded that the *Bacillus* (*Bacillus subtilis* RJAS19 and G8, *Bacillus samyllostridium* CH86‐1 and CH51, and *Bacillus subtilis* DC27) is the main strain for producing fibrinolytic enzymes (Ren et al., 2021).

**TABLE 1:** Sources of fibrinolytic enzymes.

Sources	Strains	Name	Genus	References
Pigeon pea	*Bacillus subtilis* 14714, *Bacillus subtilis* 14715, *Bacillus subtilis* 14716, *Bacillus subtilis* 14718	Natto	*Bacillus subtilis*	Lee et al. (2015)
Natto	*Bacillus subtilis* RJAS19	Nattokinase	*Bacillus subtilis*	Kumar et al. (2014)
Douchi	*Bacillus subtilis* LD‐8547	*Subtilis* DFE	*Bacillus subtilis*	Yuan et al. (2012)
Fermented shrimp paste	*Bacillus subtilis* sp.*nov*. SK006	—	*Bacillus* sp.*nov*.	Hua et al. (2008)
Cow dung	*Bacillus subtilis* sp. IND7	IND7	*Bacillus* sp.	Vijayaraghavan et al. (2016)
Cheonggukjang	*Amyloliquefaciens* CH51	AprE51	*Amyloliquefaciens*	Kim et al. (2009)
Cheonggukjang	*Bacillus samyloliquefaciens* CH86‐1	AprE86‐1	*Bacillus samyloliquefaciens*	Lee et al. (2010)
Fermented soybean	*Bacillus subtilis* QK02	QK‐2	*Bacillus subtilis*	Ko et al. (2004)
Indonesian fermented foods	*Lactobacillus plantarum* FNCC 260	—	*Lactobacillus plantarum*	Yogeswara et al. (2020)
Natto	*Bacillus subtilis G8*	Nattokinase	*Bacillus subtilis*	Pinontoan et al. (2021)
The vegetable cheese Natto	*Bacillus subtilis* natto	Nattokinase	*Bacillus subtilis*	Sumi et al. (1987)
An Indonesian fermented soybean, moromi	*Bacillus subtilis* K2	—	*Bacillus subtilis*	Syahbanu et al. (2020)

NK was discovered in a traditional Japanese fermented food natto by Sumi et al. (1987). Compared with other thrombolytic agents, NK has obvious advantages, such as low production cost, less side effects, and high thrombolytic activity. Actually, it has a double mechanism of action to dissolve thrombus directly and indirectly. NK is also able to lower blood pressure while preventing thrombus formation. Research showed that NK was produced after 24 h of incubation with *Bacillus subtilis* in the presence of natto during fermentation of pigeon pea, and this enzyme also led to thrombolysis and improvement of systolic and diastolic blood pressure in rats orally administered with liquid extracts from pigeon pea (Lee et al., 2015). Hence, NK derived from food fermentation is promising to become a functional food and a substitute for existing thrombolytic agents. Cheonggukjang (CGJ), a traditional Korean food obtained from fermentation of cooked soybean straw, was proven to have thrombolytic effects and other benefits to human by stimulating immunoregulation of ER‐β in the presence of isoflavone (Kim et al., 2021). Currently, studies of CGJ mainly focus on the fermentation process and various active ingredients. Douchi, a traditional Chinese food derived through fermentation of the main raw material soybean or black soybean, is available in two types: dry and wet. Research has shown that dry Douchi and wet Douchi differ greatly in microflora and richness. In fact, dry Douchi samples were associated with higher pathogenicity than wet Douchi which may be caused by different microbial interactions during fermentation (Wang et al., 2021). Moreover, traditional fermented shrimp paste, which is a popular seasoning in Asian countries derived by mixing silver shrimp with salt, has been shown to have strong thrombolytic activity. Amazingly, the fibrinolytic enzymes produced from strains isolated from the fermented shrimp paste are not homologous to other existing ones and exhibit activity against pepsin and trypsin. Therefore, fermented shrimp paste has great potential for application in food fermentation and nutrition (Wong & Mine, 2004).

Furthermore, fermented foods have been proved to alter intestinal flora, but their mechanisms of action require in‐depth studies. In addition to benefits to the host, fermented foods are also able to influence the intestinal flora in the host directly through flavone and polyphenols secreted during fermentation. In order to confirm this conception, a mixture of eight soybeans commonly used in China was allowed to ferment naturally (without any microorganism) and in the presence of lactobacillus, finding that the total polyphenol content (TPC) obtained with the two fermentation methods was higher than non‐fermented foods (Leeuwendaal et al., 2022). A similar study of other soybean products reported that fermented samples had higher TPC and flavone content than non‐fermented foods (Zhai et al., 2018). In another study on a novel green soybean shuidouchi fermented by *Bacillus velezensis*, it showed that the fermented green soybean shuidouchi (FGSS) had higher contents of chemical components than the unfermented green soybean shuidouchi (UGSS). Moreover, the fibrinolytic activity of FGSS was 234.8 FU/g dry weight (DW), whereas the UGSS did not have fibrinolytic activity (Liu et al., 2021). Thus, it can be concluded that fermentation is an effective technique to improve the content of bioactive compounds in fermented soybeans and secretions from microbial strains during the fermentation process. Overall, fermentation endows foods with a higher nutritional value and plays an irreplaceable role in the field of food research in both the past and the future.

## 5 OPTIMIZATION OF THE FERMENTATION PROCESS OF MICROORGANISMS FOR FIBRINOLYTIC ENZYME PRODUCTION

Optimization of fermentation conditions is the key factor for the high production of fibrinolytic enzymes from microorganisms in solid‐state or liquid‐state fermentation. Over the past years, the strains obtained by researchers have varied and different nutritional requirements (Chaturvedi & Chakraborty, 2022; Lan et al., 2020). Therefore, it is necessary to optimize the fermentation process for each bacterium to obtain a strain with high production and activity of fibrinolytic enzymes.

During the fermentation process, the composition of the culture medium affects the strain growth, thereby leading to a difference in the activity of the same fibrinolytic enzyme. Particularly, carbon source, nitrogen source, and inorganic salt greatly affect strains with high production of fibrinolytic enzymes. Carbon source and nitrogen source are important energy sources required for microbial fermentation, including glucose, sucrose, maltose, and soluble starch among others. Research showed that compared with soluble starch, the yield of NK was up to 3523 U/mL, being 1.46‐fold higher when sucrose was used as the carbon source, and that sucrose concentration would also result in an increased or decreased production of fibrinolytic enzymes, with maximum activity 35 U/mL observed at the concentration of 4086 U/mL (Wu et al., 2019). Culture media rich in soybean protein are the main source of high production of fibrinolytic enzymes, such as peptone, beef extracts, and soybean milk. In addition to the traditional optimization methods, various statistical optimization methods have also been widely used in the optimization process of traditional fermented foods, such as single‐factor and orthogonal experiments, response surface methodology (RSM), and fractional factorial design (FFD), which can more accurately predict the optimized conditions for fermented strains and the most suitable media model for strain growth. As shown in Table 2, based on the central composite design (CCD) and response surface design (RSD), use of 1.5% maltose and 4.0% yeast extract + peptone (1:1) for incubation of *Serratia* sp. KG‐2‐1 strain yielded maximum plasmin activity of 250.40 U/mL after 24 h, indicating that the models have great potential for application in the industry.

**TABLE 2:** Traditional fermentation optimization techniques.

Strains	Optimum mediums	Optimization techniques	Activity predicted	Activity observed	References
*Bacillus cereus* IND1	80% (v/w) moisture, 0.2% (w/w) beef extract, and 0.0568% sodium dihydrogen phosphate	Two‐level full‐factorial design, a central composite design (CCD), and Response surface methodology (RSM)	3750 ± 118 U/mL	3670 ± 48 U/mL	Vijayaraghavan and Vincent (2014b)
*Pseudoalteromonas* sp. IND11	pH 8.0, 120% moisture, 0.1% maltose, 0.5% casein, and 0.25% sodium dihydrogen phosphate	CCD and RSM	1340 U/mL	1365 U/mL	Vijayaraghavan and Vincent (2014a)
*Bacillus* sp. IND12	109.73% moisture, 0.57% sucrose, and 0.093% MgSO_4_	Full‐factorial design, two‐level full‐factorial design, central composite rotational design (CCRD) and RSM	4168.68 ± 36.4 U/g	4143 ± 12.31 U/g	Vijayaraghavan et al. (2017)
*Paenibacillus* sp. strain‐IND8	Sucrose (0.5%), NaH_2_PO_4_ (0.075%), and moisture (113.64%)	One factor at a Time and Fractional factorial design (FFD) CCD and RSM	4720 U/mL	4683 U/mL	Vijayaraghavan and Prakash Vincent (2014)
*Serratia* sp. KG‐2‐1	1.5% maltose, 4.0% yeast extract + peptone (1:1), 24 h incubation time, and neutral pH	CCD and RSM	243.75 U/mL	250.40 U/mL	Taneja et al. (2017)
*Bacillus subtilis* WR350	35 g/L sucrose, 20 g/L corn steep powder and 2 g/L MgSO_4_·7H_2_O	Single‐factor and orthogonal experiments	1573 U/mL	1610 U/mL	Wu et al. (2019)

It was reported that the presence of acetylurea as a by‐product in the fermentation process was detected by Xiao et al. (2022) while optimizing carbon and nitrogen sources for NK production. This substance could effectively improve the production of NK in a fermentation tank containing carbon and nitrogen sources. However, the presence of an acetylurea protein band was not found in the supernatant as confirmed by sequencing of the *aprN* gene of NK and SDS‐PAGE analysis. Therefore, the application of acetylurea in industrial production of fibrinolytic enzyme still requires further validation. In order to improve the industrial production of fibrinolytic enzyme (Liu, Zheng, et al., 2019), arranged multiple promoters in tandem in *Bacillus subtilis* WB800 to enhance the expression level of NK, finding that the highest NK production reached 264.2 ± 7.0 FU/mL, which was mediated by the triple promoter system P (PHpaII‐PHpaII‐PP43). Besides, different agricultural residues may be utilized as carbon source for solid‐state fermentation (SSF), for example, addition of yeast extract to whey cheese‐containing fermentation media resulted in an increased activity of fibrinolytic enzyme from 789.93 to 833.43 U/mL (Sahoo et al., 2020). However, considering that the enzymatic activity and stability of fibrinolytic enzymes are influenced by temperature and pH, purification and characterization of fibrinolytic enzymes from different sources are warranted to ensure maximum recovery of the enzymes with low cost and high activity (Taneja et al., 2017).

## 6 EXTRACTION AND PURIFICATION OF FIBRINOLYTIC ENZYME

Although the production of fibrinolytic enzyme has been greatly improved by optimizing the fermentation techniques, the recovered fibrinolytic enzyme is usually not pure enough due to the presence of nutrients and other proteins. Therefore, isolation and purification of the fibrinolytic enzyme to improve the purity and recovery are critically important for further functional studies. The purification method varies depending on the sources of fibrinolytic enzymes (as shown in Table 3). For example, precipitation and chromatographic approaches for purification of *Bacillus cereus* RSA1 resulted in 33.1% recovery of proteins with 2.3‐fold purification (Sharma et al., 2019). However, three‐phase partitioning (TPP) leads to significantly higher purification and activity recovery than other purification technologies, such as salting out of proteins using (NH_4_)_2_SO_4_ precipitation and ion‐exchange chromatography. According to Romy Garg and Bhaskar N (Garg & Thorat, 2014), TPP used to purify NK from fermentation broth of *Bacillus natto* NRRL‐3666 resulted in an activity recovery rate of up to 129.5%, approximately 4 times that of NK from *Bacillus cereus* RSA1. In addition, recombinant proteins were also purified using strategies based on the CTAB/isooctane/hexanol/n‐butyl alcohol reverse micellar system, as well as a combination of affinity chromatography and affinity labeling (Tripathi & Shrivastava, 2019; Zhou et al., 2022).

**TABLE 3:** Purification methods for fibrinolytic enzymes.

Sources	Purification methods	Recovery (%)	Purification fold (folds)	References
*Bacillus velezensis* SN‐14	CTAB/isooctane/hexyl alcohol/n‐butyl alcohol reverse micellar system	44.5 ± 1.9	4.9 ± 0.05	Zhou et al. (2022)
*Bacillus subtilis* BK‐17	Gel‐filtration and ion‐exchange chromatography	29.0	929.0	Jeong et al. (2001)
*Bacillus amyloliquefaciens* DC‐4	(NH_4_)_2_SO_4_ (30%–60%); Dialysis CM‐Sepharose FF column	2.8	11.5	Peng et al. (2003)
*Bacillus* sp. strain DJ‐4	DEAE‐Sepharose CL‐6B column, TSK‐gel filtration, Toyopearl HW55 FF	0.7	6.8	Kim and Choi (2000)
*Bacillus amyloliquefaciens* MJ5‐41	Chromatographic methods CM‐Sepharose cation‐exchange chromatography, ultrafiltration, Sephadex G‐100 gel filtration	6.3	5.0	Jo et al. (2011)
*Bacillus subtilis* A26	Ultrafiltration, Mono Q‐Sepharose anion exchange chromatography	12.7	7.5	Agrebi et al. (2009)
*Bacillus subtilis* DC33	(NH_4_)_2_SO_4_ precipitation, Phenyl Sepharose 6FF DEAE‐Sepharose FF, Sephadex G50 gel filtration	13.0	34.6	Wang et al. (2006)
*Bacillus subtilis* TP‐6	Ammonium sulfate fractionation, SP sepharose cation‐exchange chromatography	16.0	200.0	Kim et al. (2006)

For intracellularly expressed fibrinolytic enzymes, recombination of the target gene with vector DNA is commonly used for overexpression in prokaryotic systems. Recombinant DNA is replicated and amplified in a variety of bacterial hosts, commonly including *Escherichia coli*, *yeast*, and *Bacillus licheniformis* (Guangbo et al., 2021; Liang et al., 2007; Wei et al., 2015). For engineered strains, further purification methods should be considered, such as Ni‐NTA and His‐tagged affinity chromatography using a fast protein liquid chromatography system to purify recombinant NK, which not only overcomes the limitations of traditional methods but also presents high recovery of fibrinolytic enzymes (Modi et al., 2023). In addition to affinity‐labeled purification methods, separation by magnetic beads has been suggested as a suitable alternative. This study demonstrated Ni^2+^ charged superparamagnetic silica nanoparticles have specific affinity toward 6xHistidine‐tagged recombinant protein, as well as being a highly effective, economic, biocompatible, and flexible approach (Mohapatra et al., 2007).

## 7 PHYSICOCHEMICAL PROPERTIES OF FIBRINOLYTIC ENZYMES

The physical and chemical properties of fibrinolytic enzymes such as molecular weight (kDa), optimum temperature (°C), optimum pH value, metal ions, and inhibitors must be characterized well. Overall, the molecular weights of fibrinolytic enzymes isolated and purified from traditional fermented foods generally vary from 28.0 kDa to 30.0 kDa, except for KSK‐II and SK006. Most of the fibrinolytic enzymes display an optimal fibrinolytic activity under neutral to weakly alkaline (pH 7.0–9.0) conditions. It has been reported that AprE51, *subtilisin* DJ‐4, and KSK‐II have high activity under acidic or extremely alkaline conditions (Kim et al., 2009; Kim & Choi, 2000; Kotb, 2015). However, a novel neutral protease purified from fermented shrimp paste displayed an optimal activity under acidic to neutral (pH 3.0–7.0) conditions, with an average optimal temperature range of 30.0–50.0°C (Wong & Mine, 2004). The maximum and minimum optimal temperatures were 55.0°C (fibrinolytic enzyme derived from strain *Bacillus subtilis* DC33) and 30.0°C (fibrinolytic enzyme derived from strain *Bacillus* sp. *nov*. SK006) (Hua et al., 2008; Wang et al., 2006).

Metal ions and some inhibitors play a very important role in the catalytic activity of fibrinolytic enzymes. As shown in Table 4, metal ions such as monovalent K^+^, divalent Zn^2+^, Fe^2+^, Ni^2+^, Mn^2+^, Ca^2+^, and trivalent Fe^3+^ and Al^3+^ can activate or inhibit the fibrinolytic activity to a varying extent. In addition to metal ions, different types of protease inhibitors, including phenylmethylsulfonyl fluoride (PMSF), ethylenediaminetetraacetic acid (EDTA), p‐chloromercuryl benzoate (PCMB), soybean trypsin inhibitor (SBTI), and L‐lysyl chloromethane hydrochloride (TLCK), can also affect the fibrinolytic activity. Protease inhibitors can be classified into three types according to their effect on enzyme activity. The first type is the commonly used protease inhibitor PMSF, which, in the presence of 1 mM SBTI, could completely inhibit the amidolytic activity of *subtilisin* FS33, resulting in a complete loss of enzyme activity (Wang et al., 2006). The second type is the metalloproteinase inhibitor EDTA, which could significantly inhibit the enzyme activity of purified KSK‐II, but returned to normal under the action of the metal ion Fe^2+^ (Kotb, 2015). The third type is the inhibitors of serine metalloproteinases, which can be inhibited by both a serine protease inhibitor and a metalloproteinase inhibitor. For example, the enzyme activity of a fibrinolytic enzyme purified from Chlorella vulgaris was enhanced in the presence of Fe^2+^ and inhibited by PMSF and EDTA (Silva et al., 2018).

**TABLE 4:** Physicochemical properties of fibrinolytic enzymes.

Sources	Fibrinolytic enzyme	Mass (kDa)	Optimal pH	Optimal temperature (°C)	Activators	Inhibitors	References
*Bacillus subtilis* DC33	*Subtilisin* FS33	30.0	8.0	55.0	—	PMSF, SBTI	Wang et al. (2006)
*Bacillus amyloliquefaciens* Jxnuwx‐1	—	29.0	7.6	41.0	—	PMSF, EDTA, SBTI, Fe^3+^, Fe^2+^	Yang et al. (2020)
*Amyloliquefaciens* DC‐4	*Subtilisin* DFE	28.0	9.0	48.0	—	Hydrochloride, Leupeptin, Pepstatin A	Peng et al. (2003)
Fermented shrimp paste	—	18.0	3.0–7.0	30.0–40.0	—	EDTA, Cu^2+^	Wong and Mine (2004)
*Bacillus* sp. *nov*. SK006	—	43.0–46.0	7.2	30.0	Zn^2+^	PMSF, EDTA, PCMB, Cu^2+^, Ca^2+^, Fe^3+^, Hg^2+^	Hua et al. (2008)
*Bacillus subtilis* natto B‐12	B‐12 nattokinase	29 ± 0.3	8.0	40.0	Zn^2+^	Fe^3+^, Al^3+^	Wang et al. (2009)
Bile salt‐tolerant nattokinase	*Bacillus mojavensis* LY‐06	29.0	8.0	50.0	Ba^2+^, Ni^2+^, K^+^, Mg^2+^	Mn^2+^, Ca^2+^, Fe^3+^	Li et al. (2022)
*Bacillus* sp. strain DJ‐4	*Subtilisin* DJ‐4	29.0	10.0	40.0	—	PMSF, Cu^2+^, Zn^2+^	Kim and Choi (2000)
*Lactobacillus plantarum* KSK‐II	KSK‐II	43.6	10.0	50.0	Fe^2+^	EDTA	Kotb (2015)
*Bacillus amyloliquefaciens* FCF‐11	FCF‐11	18.2	8.0	40.0	Fe^2+^, Mn^2+^	SBTI, TLCK, PMSF, Aprotinin	Kotb (2014)
*Bacillus amyloliquefancies* CH51	AprE51	27.0	6.0	45.0	—	PMSF, EDTA	Kim et al. (2009)

## 8 THROMBOLYTIC EFFECTS IN VIVO

In recent years, in order to evaluate the thrombolytic effects of fibrinolytic enzymes in vivo and improve the efficacy and safety of fibrinolytic therapy, many studies have been conducted on fibrinolytic enzymes derived from traditional fermented foods. The fibrinolytic enzymes isolated from Douchi (a soybean‐fermented food in China) and natto have received much attention.

Douchi fibrinolytic enzyme (DFE) from *Bacillus subtilis* LD‐8547 was studied in vivo by Yuan et al. (Yuan et al., 2012). Acute toxicity assay, thrombolytic effects on carrageenan‐induced thrombosis model, effects on the bleeding and clotting time, lytic effects on whole blood clots and plasma clots, as well as effects on the model of arterial thrombosis induced by ferric chloride were performed. The results showed that DFE had no obvious acute toxicity to mice, and there were no abnormal changes in the pathological sections from the hearts, livers, spleens, lungs, kidneys, stomachs, and intestines of all mice. DFE significantly prevented tail thrombosis in the carrageenan‐induced thrombosis model in mice as indicated by measuring the length of the tail thrombus. Meanwhile, DFE also had an effect on the bleeding and clotting time in the mice tested, and the duration of effect was positively correlated with DFE in a dose‐dependent manner. In addition, DFE could lyse whole blood clots as well as plasma clots within 30 minutes. Whereas in the model of arterial thrombosis induced by ferric chloride, the results demonstrated that although the thrombus‐formed vessel wall was a bit darker than normal vessel after DFE injection 2d, the thrombus‐formed vessel then became bright red and the blood flowed normally again.

In another model of thrombosis established by Xu et al. (2014), the activity of NK in vivo was demonstrated. K‐carrageenan was injected subcutaneously into the toes of Sprague–Dawley (SD) rats, which were then treated with varying doses of NK after thrombosis was confirmed, and vermis kinase was used as a positive control. The resulting thrombolysis was histologically assessed. The results revealed partial thrombolysis in the tail vessels of rats treated with NK or vermis kinase, and higher FDP and D‐dimer levels in rats treated with high‐dose NK than in those treated with normal saline, indicating that NK exerted thrombolytic effects in vivo.

In a set of human trials including healthy volunteers, patients with cardiovascular risk factors and patients on dialysis, two NK capsules (2000 FU/capsule) were orally administered. After 2 months of administration, a sustained decrease in all three factors – fibrinogen, factor VII, and factor VIII – was observed in all groups. No adverse effects were noted in any subjects, with the heart rates, body weight, and uric acid levels remaining stable (Hsia et al., 2009).

## 9 FUNCTIONAL PROPERTIES OF FIBRINOLYTIC ENZYMES

During the fermentation of traditional fermented foods, a variety of active ingredients are produced by microorganisms with properties of thrombolytic, anti‐coagulant, anti‐hypertensive, anti‐inflammatory, or neuroprotective properties (Table 5). NK, a potent thrombolytic agent, has been shown to have anti‐atherosclerotic and anti‐hyperlipidemia effects. In a clinical study involving 1062 participants, NK at a dose of 10,800 FU/day was found to have effectively managed the progression of atherosclerosis and hyperlipidemia, along with a significant reduction observed in the thickness of the carotid artery intima‐media and the size of the carotid plaque (Chen et al., 2022). Another study reported a significant increase in fibrin/fibrinogen degradation products (thrombolysis and anti‐coagulation) 4 h after NK administration in 12 subjects who received a single dose of NK 2000 FU or placebo, followed by a group crossover (Kurosawa et al., 2015). Thus, based on the above, fibrinolytic enzymes from traditional fermented foods can be used as effective thrombolytic agents/anti‐coagulants to reduce the risk of thrombosis in humans.

**TABLE 5:** Functional properties of fibrinolytic enzymes.

Fermented food	Active ingredient	Function	References
Soybean	Phenolic, flavonoid	Neuroprotection	Kang et al. (2016)
Tempeh	GABA and anthocyanin	Neuroprotection	Hwang et al. (2019)
Fermented soybean	Nattokinase	Anti‐inflammatory	Wu et al. (2020)
Fermented beans	GABA	Anti‐hypertensive	Suwanmanon and Hsieh (2014)
Milk	Angiotensin I‐converting enzyme Inhibitor (ACEI), GABA	Anti‐hypertensive	Liu et al. (2011)
Natto	Crude extracts of NK (NCE)	Anti‐inflammatory	Zhang et al. (2021)
Natto	Crude extracts of NK (NCE)	Anti‐cancer	Yan et al. (2019)

## 10 IMPROVEMENT OF THE BIOLOGICAL VALUE OF FIBRINOLYTIC ENZYMES

Based on the physiochemical properties of fibrinolytic enzymes summarized in Table 4, most fibrinolytic enzymes exhibit optimal fibrinolytic activity under neutral to weakly alkaline conditions, whereas a few enzymes lose their fibrinolytic activity completely in acidic conditions. Therefore, several approaches such as molecular modification, enzyme immobilization, and construction of a functional delivery system can be utilized to improve the stability and biological value of fibrinolytic enzymes.

The efficacy of purified fibrinolytic enzymes after oral administration is limited in an acidic condition of gastric juice. It was reported that the functional delivery system protected fibrinolytic enzymes from damage by gastric juice and ensured maximum stability of the enzymes. In 2017, the method of nanoencapsulation with a polylactic acid‐co‐glycolic acid (PLGA) copolymer was tested for NK, which was found to penetrate the blood–brain barrier while maintaining its enzymatic activity and delayed the progression of Alzheimer's disease by suppressing the Aβ_40_ plaques (Bhatt et al., 2017). In 2019, the technique of multi‐site‐directed mutagenesis was utilized to improve the enzymatic properties of NK such as acid resistance and thermal stability, and the molecular mechanism of increased stability of mutants S78T and Y217K under acidic conditions was clarified (Liu et al., 2019). In 2020, the concept of chitosan‐based microparticles with a bilayer shell–core structure of NK encapsulated with chitosan as the inner core and casein as the outer protective shell was proposed. To realize this concept, casein was cross‐linked to chitosan itself and NK on the core of chitosan utilizing the activity of the protein cross‐linker transglutaminase (TG) to construct a bilayer protective shell. The results demonstrated the feasibility of using chitosan‐based microparticles as a functional delivery system (Zhang et al., 2020).

In addition to optimization of the drug delivery system and molecular modification, fibrinolytic enzymes with higher activity can also be obtained by improving the storage stability of the enzymes themselves. For example, spray‐drying of microencapsules was formed upon encapsulation of substances of a varying molecule size by controlling the mesh size (Li et al., 2021; Rezvankhah et al., 2020). They not only help to protect the fibrinolytic enzyme from being dissolved in the environment of gastric juice but also ensure maximum ambient storage stability of the NK powder samples close to that in a neutral pH environment. In future research and development, the functional delivery system may be a potential candidate that will better promote the application of fibrinolytic enzymes in the medical and/or food industry. However, further in‐depth studies are required to address whether this system will lead to degradation of the fibrinolytic enzymes, and how long the fibrinolytic activity will be maintained in the delivery route.

## 11 CONCLUSION

As the population of China is increasing, there has been a general rise in the morbidity and mortality because of CVDs. Therefore, finding novel fibrinolytic agents has become particularly important. Over the past years, extensive studies have been conducted on the production of microbial fibrinolytic enzymes from traditional fermented foods to isolate novel enzymes from the production of traditional fermented foods of varying sources. For the sustainable industrialization of fibrinolytic enzymes, optimizing the fermentation conditions not only helps to save the production cost but also improves the productivity of fibrinolytic enzymes. These novel fibrinolytic enzymes were purified and measured for molecular weight to investigate the effects of pH, temperature, charged ions, and inhibitors on their physicochemical properties. In addition, fibrinolytic enzymes were also studied for their thrombolytic effects in vitro and in vivo. Although the production of fibrinolytic enzyme from traditional fermented food has great potential for development, the fermentation of traditional food is still facing many urgent problems yet to be solved. First of all, on the basis of optimizing fermentation conditions to achieve high production of fibrinolytic enzymes, the most important thing is to produce fibrinolytic enzymes with high activity and high recovery rate. Second, although there are many reports about the thrombolytic effects of fibrinolytic enzymes in vitro or in vivo in recent years, issues regarding their safety, mode of administration, and stability are still needed to be further studied. Finally, the mechanism of action and pharmacokinetics of fibrinolytic enzymes need to be further investigated through clinical experiments and human trials.

## AUTHOR CONTRIBUTIONS

**Panpan Wang:** Conceptualization (equal); writing – original draft (equal). **Cuiying Peng:** Writing – review and editing (equal). **Xiaomei Xie:** Writing – review and editing (equal). **Xiongwei Deng:** Writing – review and editing (equal). **Meizhi Weng:** Conceptualization (equal); writing – review and editing (lead).

## FUNDING INFORMATION

This work was supported by the National Natural Science Foundation of China Project (82060699, 82060709); Distinguished Youth Foundation Project of Jiangxi Natural Science Foundation (20192ACBL21032); General Project of Jiangxi Natural Science Foundation (20224BAB206103, 20192BAB205098, S2023ZRMSL1104); Youth Project of Jiangxi Natural Science Foundation (20171BAB215061); Innovation and Entrepreneurship Project (202210412372, S202310412086).

## CONFLICT OF INTEREST STATEMENT

The authors declare that they have no competing interest.

## ETHICS APPROVAL

There is no need for ethical approval as this was a review article.

## Data Availability

Data sharing is not applicable to this article as no datasets were generated or analyzed during the current study.
